# A Solution-Processable, Nanostructured, and Conductive Graphene/Polyaniline Hybrid Coating for Metal-Corrosion Protection and Monitoring

**DOI:** 10.1038/s41598-017-15552-w

**Published:** 2017-11-09

**Authors:** Saerona Kim, Thanh-Hai Le, Chul Soon Park, Geunsu Park, Kyung Ho Kim, Semin Kim, Oh Seok Kwon, Gyun Taek Lim, Hyeonseok Yoon

**Affiliations:** 1 School of Polymer Science and Engineering, Chonnam National University, 77 Yongbong-ro, Buk-gu, Gwangju, 61186 South Korea; 2 Department of Polymer Engineering, Graduate School, Chonnam National University, 77 Yongbong-ro, Buk-gu, Gwangju, 61186 South Korea; 3 BioNanotechnology Research Center, Korea Research Institute of Bioscience and Biotechnology (KRIBB), 125 Gwahak-ro, Yuseong-gu, Daejeon, 34141 South Korea

## Abstract

A smart and effective anticorrosive coating consisting of alternating graphene and polyaniline (PANI) layers was developed using top-down solution processing. Graphite was exfoliated using sonication assisted by polyaniline to produce a nanostructured, conductive graphene/polyaniline hybrid (GPn) in large quantities (>0.5 L of 6 wt% solution in a single laboratory-scale process). The GPn was coated on copper and exhibited excellent anticorrosion protection efficiencies of 46.6% and 68.4% under electrochemical polarization in 1 M sulfuric acid and 3.5 wt% sodium chloride solutions, chosen as chemical and seawater models, respectively. Impedance measurements were performed in the two corrosive solutions, with the variation in charge transfer resistance (*R*_ct_) over time indicating that the GPn acted as an efficient physical and chemical barrier preventing corrosive species from reaching the copper surface. The GPn-coated copper was composed of many PANI-coated graphene planes stacked parallel to the copper surface. PANI exhibits redox-based conductivity, which was facilitated by the high conductivity of graphene. Additionally, the GPn surface was found to be hydrophobic. These properties combined effectively to protect the copper metal against corrosion. We expect that the GPn can be further applied for developing smart anticorrosive coating layers capable of monitoring the status of metals.

## Introduction

Metal corrosion is a major problem in many industries, and it remains a popular research topic in materials science, with emerging applications^1–4^. In general, corrosion consists of the irreversible deterioration of metals resulting from chemical, or often electrochemical, reactions induced by the surrounding environment. Material properties such as appearance, chemical reactivity, mechanical strength, and electrical conductivity are significantly affected by corrosion. In particular, metallic corrosion in key conducting components, such as conductors and electrodes in devices, can cause unexpected and serious failure in device performance. It is also important to note that the corrosion of electrical/electronic components is insidious and cannot be easily detected in sophisticated devices. A rich variety of anti-corrosive coatings consisting of small molecules, polymers, carbon species, oxides, and alloys are available^5–15^. Anticorrosive mechanisms involve barrier, inhibitive, and galvanic effects, depending on the nature of the anti-corrosive coating material^5,6^. In most cases, these coatings render the metal surface chemically impermeable and/or electrically insulating to prohibit undesirable chemical/electrochemical reactions.

Conducting polymers have been investigated for their ability to suppress corrosion reactions, taking advantage of their inherent reversible redox activity^6,16^. While insulating coating agents inhibit the flow of corrosion current, conducting polymers allow current flow at the metal surface. The redox-reaction-based conductivity of conducting polymers involves introducing/releasing dopant ions^17^, which can provide a unique protective mechanism against corrosion. A plausible protective mechanism for conducting polymers includes anodic protection and/or a barrier effect, which is highly dependent on the dopant ions^18^. Polypyrrole and polyaniline (PANI) are the main conducting polymers that have been investigated for corrosion control^18–20^. The greatest benefit of using conducting-polymer coatings could be their electrical conductivity and electrochemical properties, such as capacitance and impedance, which makes them a unique and versatile class of anticorrosive coating agents.

Herein, we examine the ability of a nanostructured, conductive graphene/polyaniline hybrid (GPn) coating to protect a metal against corrosion. Graphene sheets were physically exfoliated using soluble PANI in an organic solvent, resulting in a graphene dispersion stabilized by PANI. The GPn dispersion exhibited high colloidal stability and was readily coated as a thin film on copper metal, with the coating consisted of alternating graphene and PANI layers. The high conductivity of graphene contributed to an increase in the PANI conductivity, which strengthened the anticorrosive behavior of the GPn. Furthermore, the two-dimensional graphene layers provide a physical barrier against the migration of corrosive species^21–25^, which can synergistically couple with a PANI barrier effect^26–28^. Lastly, the GPn coating allowed nondestructive, electrochemical monitoring of the metal-surface status.

## Results and Discussion

**Figure 1:**
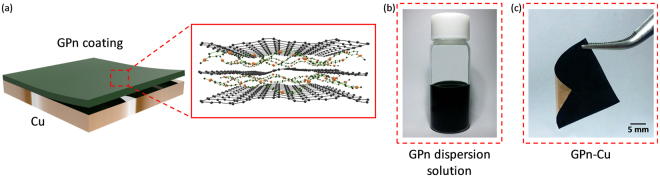
(**a**) Schematic illustration of the alternating layered structure of GPn and photos showing (**b**) the colloidal stability of the GPn dispersion and (**c**) the GPn-Cu.

A large quantity of GPn solution was first prepared (>0.5 L of 6 wt% GPn/*N*-methyl-2-pyrrolidone (NMP) solution) via a single production process using an efficient physical approach (Fig. 1). Briefly, graphite was exfoliated using sonication assisted by PANI in NMP to obtain a dispersion of graphene sheets stabilized by PANI, which could then be coated on a metallic substrate^29,30^. Owing to their two-dimensional sp^2^ carbon bonding, the graphene sheets stacked parallel to the substrate face, with PANI located between the individual graphene sheets. In other words, the GPn coating on the metallic substrate possessed a random-alternating layered graphene and PANI structure. Raman spectroscopy confirmed that the GPn coating consisted of few-layer graphene sheets and PANI^29^. Importantly, the graphene and PANI both retained their inherent electrical and electrochemical properties. The four-point-probe conductivity of the GPn was ~1.0 S cm^−1^, which is an order of magnitude higher than the value for pure PANI (~0.5 S cm^−1^), indicating that the graphene sheets acted as a conductive filler and formed conducting networks in the hybrid. In addition, graphene can act as a two-dimensional structural barrier and PANI possesses chemical properties originating from its unique conjugated system. These characteristics of the GPn coating can elicit a synergistic effect between the graphene and PANI that limits metal corrosion. Copper corrosion is mediated by electron-exchange reactions that are accompanied by ion and small-molecule transport. Electrons released from the anodic oxidation of copper are consumed by the cathodic reaction; consequently, impeding one or both anodic/cathodic reactions will retard the overall corrosion process.

**Figure 2:**
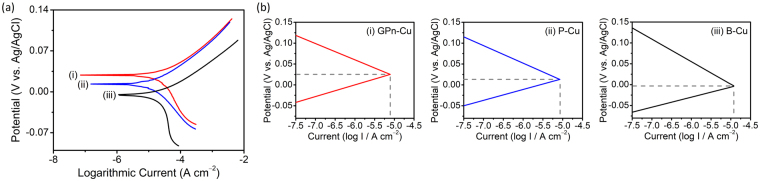
Polarization behavior of (i) GPn-Cu, (ii) P-Cu, and (iii) B-Cu in a 1 M H_2_SO_4_ solution: (**a**) Tafel plots and (**b**) Evans diagrams. P-Cu and B-Cu were used as controls.

Copper was chosen as a representative metal substrate because of its widespread use in various industries. The GPn coating showed strong attachment to the copper substrate due to the adhesive properties of PANI (Fig. S1). PANIs prepared with three additives (camphorsulfonic acid (CSA), dodecylbenzensulfonic acid (DBSA), and sorbitol) as a ternary dopant were found to have excellent adhesive properties as well as conductivity^31^. The strong adhesion of the coating layer on the metal surface is essential for practical applications. The effect of the GPn coating on copper corrosion behavior was first examined in sulfuric acid and sodium chloride solutions using potentiodynamic polarization. Bare copper (B-Cu) and PANI-coated copper (P-Cu) were employed as control samples for comparison. Representative Tafel plots of polarization curves recorded in a 1 M H_2_SO_4_ solution are presented in Fig. 2. The polarization curve clearly differs depending on the coating material. The GPn-coated copper (GPn-Cu) Tafel plot was located at a higher potential than those of B-Cu and P-Cu. In addition, the GPn coating also affected the Tafel slope. Major corrosion parameters, corrosion current density (*I*_corr_), corrosion potential (*E*_corr_), Tafel slopes (*b*_a_ and *b*_c_), polarization resistance (*R*_p_), and protection efficiency (*PE*), were calculated by extrapolating the linear sections of the Tafel plots, and are presented in Table 1. Theoretically, the corrosion current is the value of either the anodic or cathodic current at the corrosion potential (or open-circuit potential). Electrons flow from the anodic to cathodic regions during polarization, with the corrosion rate being proportional to the corrosion current density. The Tafel slope is inversely proportional to the number of electrons exchanged, which provides an insight into the kinetics of the corrosion reactions. The polarization resistance can be derived from the inverse of the polarization-curve slope, and is indicative of the resistance of copper against corrosion. From Table 1 it is clear that GPn-Cu exhibits a decreased corrosion current density, as well as an increased corrosion potential, compared with the control samples. The GPn-Cu corrosion potential is shifted to a more positive value compared with P-Cu, implying that the embedded graphene facilitated the anodic protection by PANI. Evans diagrams for the three samples are presented in Fig. 2b. The high cathodic polarization behavior of B-Cu in the sulfuric-acid solution was clearly weakened by the GPn coating. The anodic and cathodic Tafel slopes were affected by the GPn coating, indicating that the coating material affects the corrosion-reaction kinetics. Interestingly, the anodic Tafel slope increased after coating with PANI and increased again after coating with GPn, while the cathodic Tafel slope decreased. The polarization resistance of GPn-Cu was 1.9 and 1.3 times greater than that of B-Cu and P-Cu, respectively. Lastly, we calculated the protection efficiency of GPn to be 17.01% greater than that of bare PANI. These results indicate that the GPn coating serves as an excellent protective layer against copper corrosion in an acidic solution.

**Table 1:** Major corrosion parameters derived from the analysis of potentiodynamic polarization curves obtained in a 1 M H_2_SO_4_ solution.

Specimen	Parameters					
	*E*_corr_ (mV)	*I*_corr_ (*µ*A cm^−2^)	*b*_a_ (mV dec^−1^)	*b*_c_ (mV dec^−1^)	*R*_p_ (Ω cm^−2^)	*PE* (%)
GPn-Cu	30.2 ± 3.7	13.9 ± 3.8	27.9 ± 8.4	39.5 ± 4.7	511.3 ± 34.1	46.6
P-Cu	12.0 ± 2.2	17.9 ± 1.9	25.7 ± 2.3	42.1 ± 2.2	387.8 ± 16.0	29.6
B-Cu	−6.6 ± 0.7	26.5 ± 2.3	24.2 ± 1.0	53.6 ± 0.9	273.2 ± 16.2	

**Figure 3:**
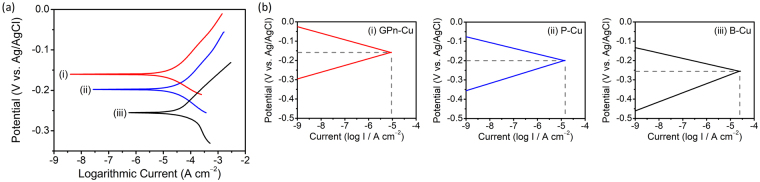
Polarization behavior of (i) GPn-Cu, (ii) P-Cu, and (iii) B-Cu in a 3.5 wt% NaCl solution: (**a**) Tafel plots and (**b**) Evans diagrams. P-Cu and B-Cu were tested as controls.

The ability of GPn to protect the copper substrate in a sodium chloride solution was also explored using the same method. Figure 3a displays Tafel plots for the three samples, B-Cu, P-Cu, and GPn-Cu, obtained through potentiodynamic polarization tests in a 3.5 wt% (0.6 M) NaCl solution. The shape of the Tafel plots recorded in the sodium chloride solution were different from those recorded in the acidic solution owing to the different corrosion mechanisms operating in these two solutions. Nevertheless, the GPn-Cu polarization curve is shifted to a higher potential compared to the P-Cu and B-Cu curves, which is similar to what was observed in the sulfuric acid solution. The major corrosion parameters were again calculated from the polarization curves and are displayed in Table 2. The corrosion current decreased significantly upon coating of the copper with GPn, while the anodic and cathodic Tafel slopes decreased and increased, respectively, in contrast to the behavior observed in the sulfuric acid solution. Evans diagrams, displayed in Fig. 3b, indicated that the GPn coating inhibited the anodically controlled corrosion of copper in the sodium chloride solution. The polarization resistance of GPn-Cu measured in the sodium chloride solution was 3.2 and 1.7 times higher than that of the control specimens B-Cu and P-Cu, respectively. Pure PANI provided a protection efficiency of 47.55% in the sodium chloride solution, while GPn provided an improved protection efficiency of 68.43%. This confirms that the GPn coating, with its alternating layered structure, was effective in protecting the copper when exposed to different corrosive environments.

**Table 2:** Major corrosion parameters derived from the analysis of potentiodynamic polarization curves obtained in a 3.5 wt% NaCl solution.

Specimen	Parameters					
	*E*_corr_ (mV)	*I*_corr_ (*µ*A cm^−2^)	*b*_a_ (mV dec^−1^)	*b*_c_ (mV dec^−1^)	*R*_p_ (Ω cm^−2^)	*PE* (%)
GPn-Cu	−159.8 ± 6.7	9.7 ± 0.8	35.4 ± 3.8	34.1 ± 5.0	775.9 ± 36.3	68.4
P-Cu	−195.0 ± 5.9	15.8 ± 1.3	38.3 ± 4.0	30.5 ± 2.3	467.0 ± 34.7	47.6
B-Cu	−242.0 ± 11.2	31.4 ± 2.5	47.1 ± 2.2	28.4 ± 5.7	244.9 ± 23.1	

**Figure 4:**
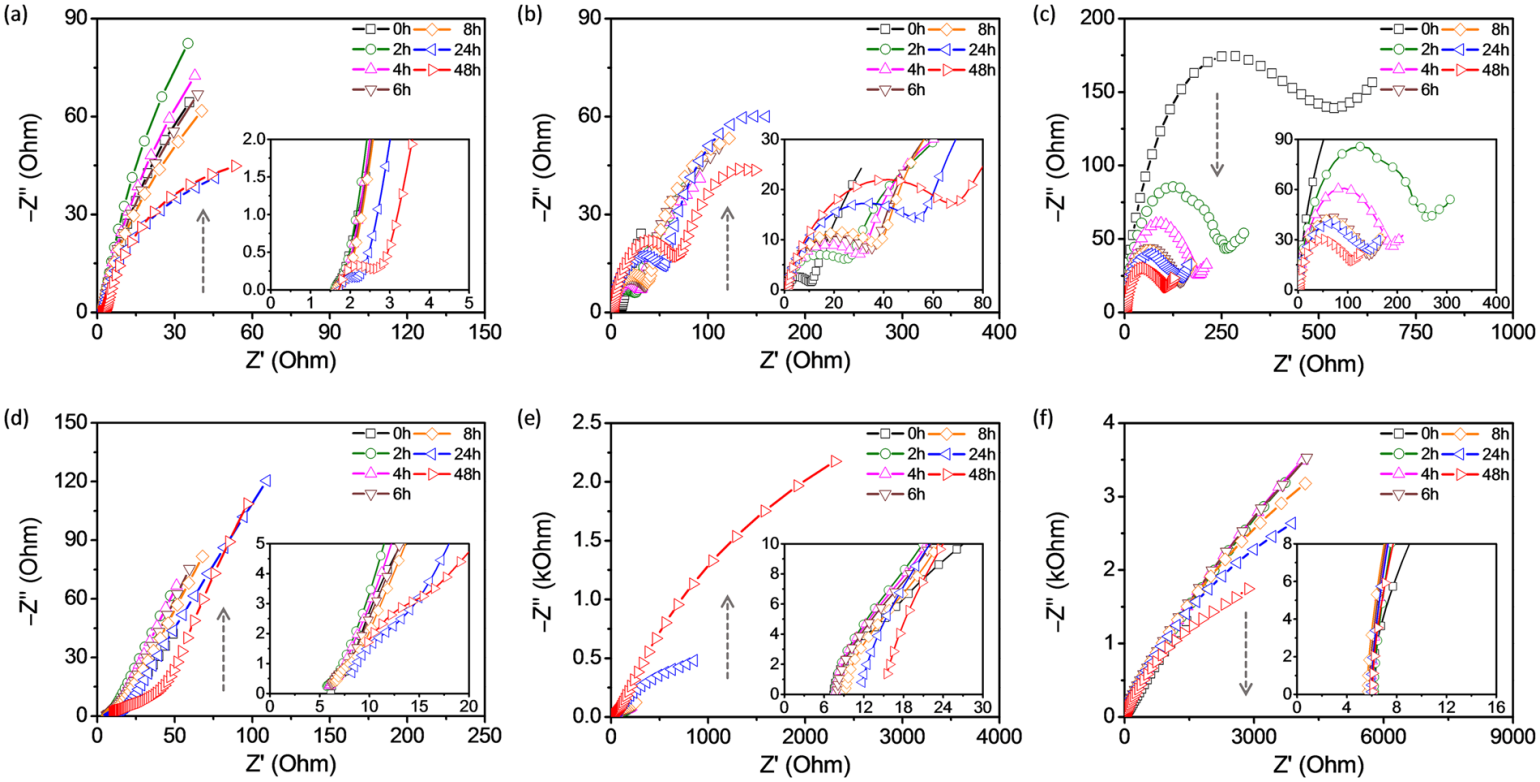
EIS Nyquist plots for (**a**,**d**) GPn-Cu, (**b**,**e**) P-Cu, and (**c**,**f**) B-Cu measured in (**a**–**c**) 1 M H_2_SO_4_ solution and (**d**–**f**) 3.5 wt% NaCl solution over a frequency range of 100 mHz to 0.1 MHz. The insets are magnified plots of the high-frequency regions.

**Figure 5:**
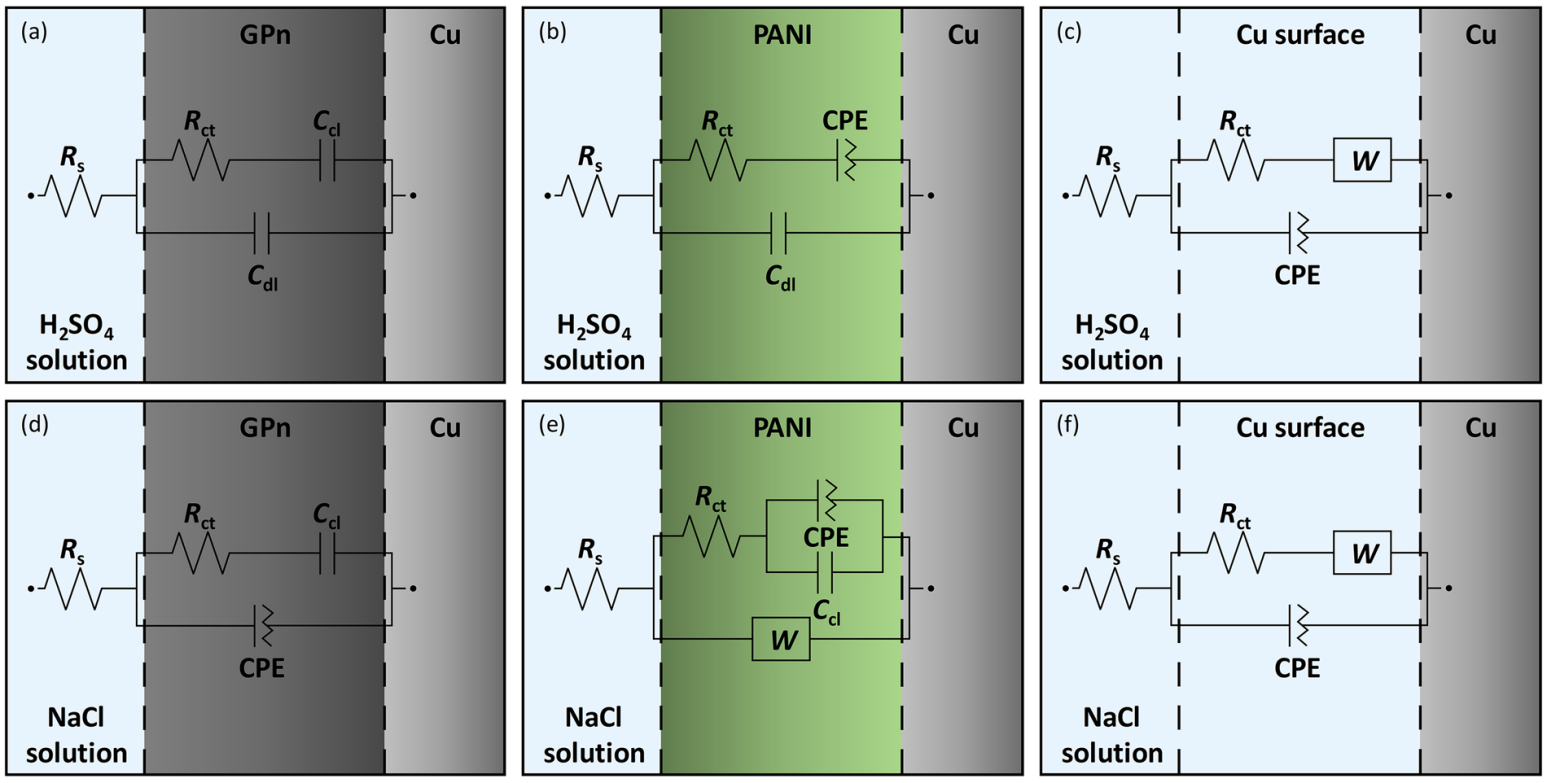
Schematics of the three electrodes, describing the distributed impedance components with equivalent circuits, in (**a**–**c**) 1 M H_2_SO_4_ solution and (**d**–**f**) 3.5 wt% NaCl solution: (**a**,**d**) GPn-Cu, (**b**,**e**) P-Cu, and (**c**,**f**) B-Cu.

**Figure 6:**
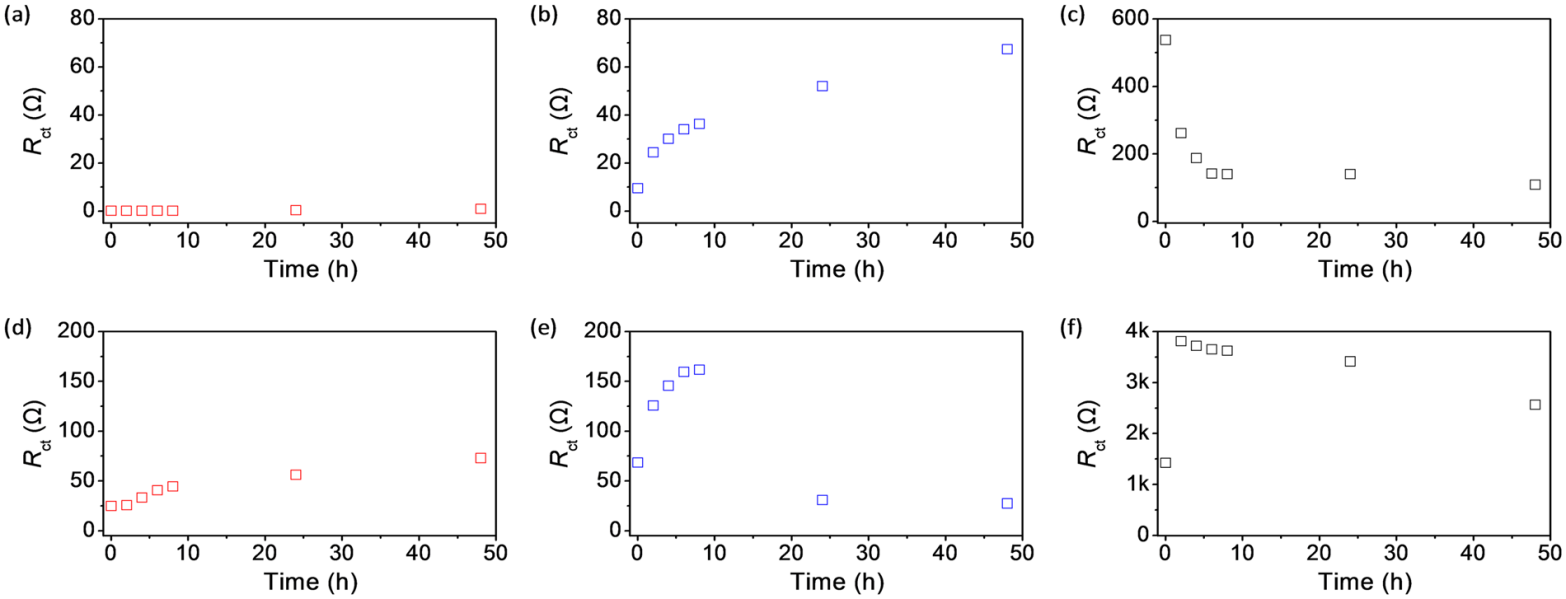
*R*_ct_ calculated by fitting the Nyquist plots to an equivalent circuit: (**a**,**d**) GPn-Cu, (**b**,**e**) P-Cu, and (**c**,**f**) B-Cu measured in (**a**–**c**) 1 M H_2_SO_4_ solution and (**d**–**f**) 3.5 wt% NaCl solution. The dots are data points and the lines are the corresponding fitting curves.

**Figure 7:**
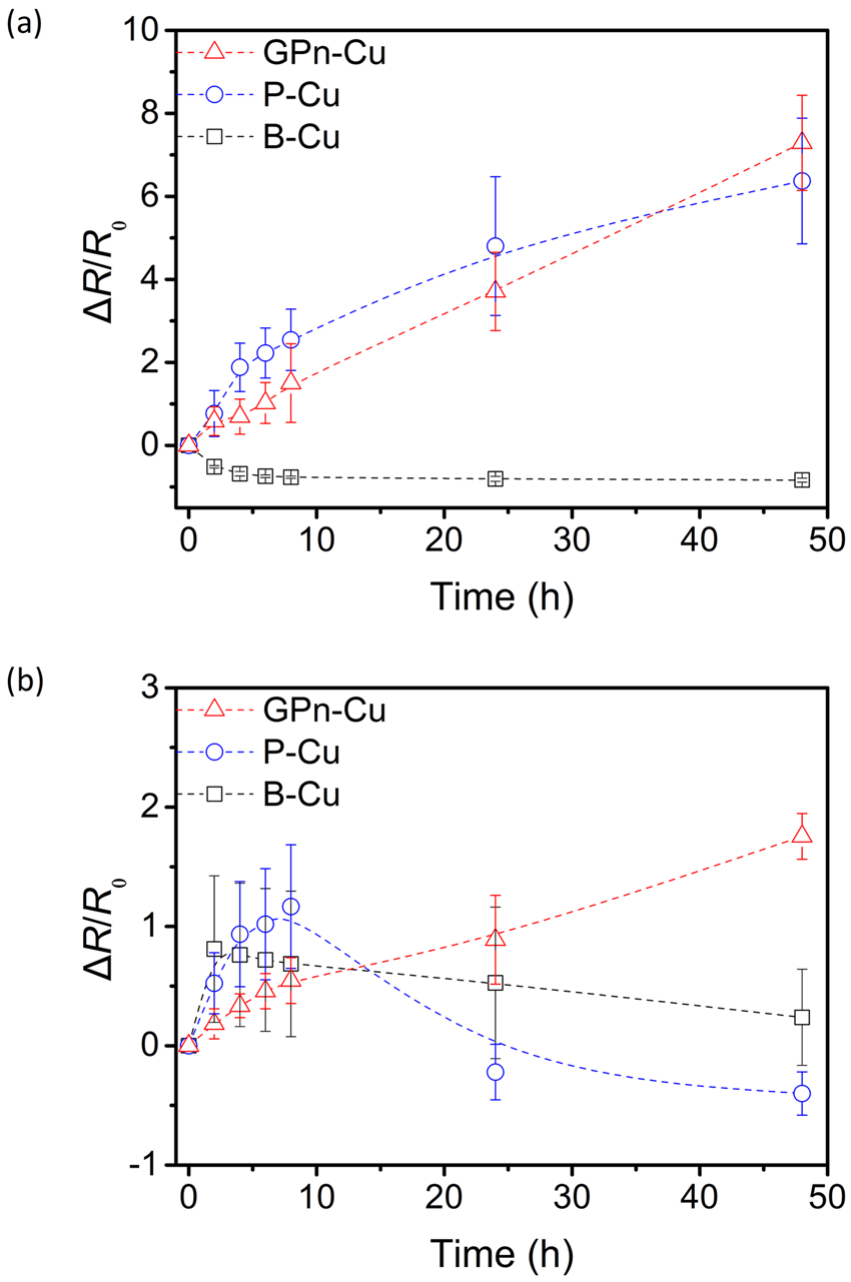
Plots of normalized *R*_ct_ as a function of exposure time in (**a**) 1 M H_2_SO_4_ and (**b**) 3.5 wt% NaCl solutions, calculated from the data in Fig. 6.

**Figure 8:**
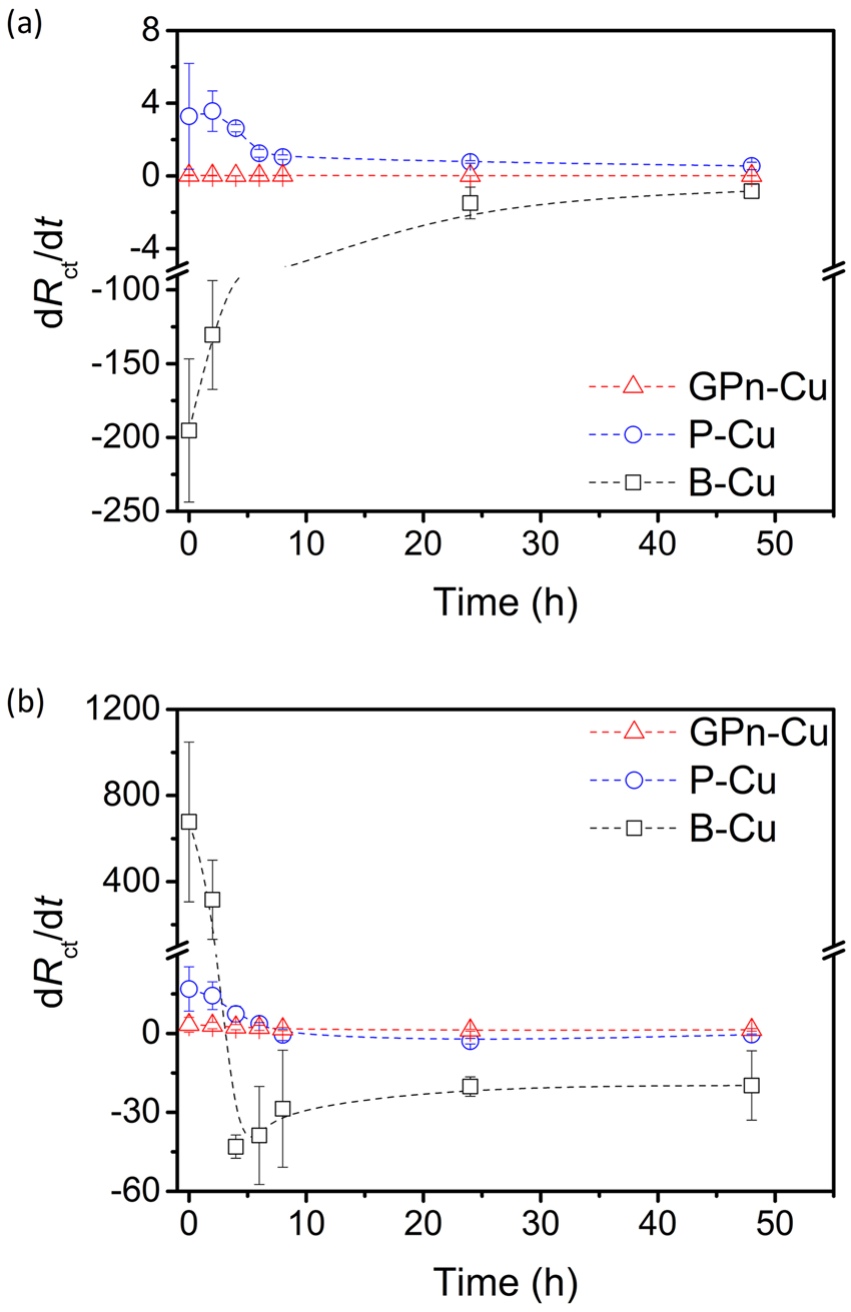
Plots of d*R*_ct_/d*t* as a function of exposure time in (**a**) 1 M H_2_SO_4_ and (**b**) 3.5 wt% NaCl solutions, calculated from the data in Fig. 6.

Electrochemical impedance spectroscopy (EIS) was used to investigate the effect of time on the copper-corrosion protection of GPn-Cu. The samples were immersed separately in 1 M H_2_SO_4_ and 3.5 wt% NaCl electrolyte solutions for 48 h. Corrosion of the copper in the samples is caused by time-dependent physical (e.g. mass transport and adsorption) and chemical (e.g. charge interactions and oxidation/reduction) processes, including i) water uptake, ii) penetration/diffusion of corrosion-related ionic and molecular species through the coating layer; and iii) formation and diffusion of corrosion products. Figure 4 shows Nyquist plots of the imaginary impedance component (Z″) against the real impedance component (Z′) at different immersion times for each sample in the two electrolyte solutions. The evolution of the Nyquist plots over time shows the effect of exposure time on the impedance. The Nyquist-plot shape is also strongly dependent on the type of coating layer as well as the electrolyte. It should be noted that the overall impedance ranges appear to be completely different for all three samples, with GPn-Cu exhibiting the lowest impedance values. Moreover, the evolution of the B-Cu impedance over time was opposite of those for GPn-Cu and P-Cu (illustrated by the arrows in Fig. 4). An equivalent-circuit model was developed for each sample (Fig. 5) to extract useful information from the Nyquist plots and further understand how corrosion occurs in these systems. In both solutions, the B-Cu Nyquist plot was fitted to a Randles circuit with an additional Warburg element, which is identical to previously reported equivalent circuits for such systems. On the other hand, the GPn-Cu and P-Cu circuit models were dependent on the immersing solution. In the sulfuric acid solution, the P-Cu circuit model was comprised of solution resistance (*R*_s_), charge transfer resistance (*R*_ct_), capacitance (*C*_cl_), and constant phase element (CPE) components, while two additional circuit components, double-layer capacitance (*C*_dl_) and a Warburg element (*W*), were used for the sodium chloride solution. *C*_cl_ and *C*_dl_ are the capacitance of the coating layer and the double-layer capacitance on the copper surface, respectively, while the CPE describes non-ideal capacitance behavior. Four circuit components, *R*_s_, *R*_ct_, *C*_cl_ and *C*_dl_, were used in the GPn-Cu sulfuric acid system. However, in the sodium chloride solution, a CPE was used in place of *C*_dl_ to model the capacitance of the GPn layer. Values for all equivalent-circuit components were calculated through fitting (Tables S1–S6). The *C*_cl_, *C*_dl_, and CPE values are interrelated, while *R*_ct_ is an independent component that is common to the equivalent circuits of the systems studied here, thus allowing direct comparison of the specimens. Figure 6 plots the calculated *R*_ct_ as a function of the exposure time. Normalized *R*_ct_ changes are also plotted in Fig. 7, clearly showing the shifting profile of *R*_ct_ as a function of the exposure time. In the sulfuric acid solution, the B-Cu *R*_ct_ initially decreases gradually from 540 to 100 Ω over the first 10 h and then levels off. The thin oxide layer on the B-Cu surface is likely etched away in the acidic solution, resulting in the decreased *R*_ct_. The initial *R*_ct_ for GPn-Cu and P-Cu is three and two orders of magnitude lower, respectively, than that of B-Cu and increases with exposure time over 50 h. Remarkably, GPn-Cu still exhibited a low *R*_ct_ (on the order of 10^−1^ Ω) after 48 h, while the P-Cu *R*_ct_ increased to an order of 10^1^ Ω. This indicates that GPn-Cu plays a role in preserving the initial surface characteristics of copper in the sulfuric acid solution. The overall *R*_ct_ trends for the systems were different in the sodium chloride solution. The corrosion behavior of copper in the sodium chloride solution is clearly different to that in the sulfuric acid solution. For example, chloride ions from sodium chloride can form copper chloride on the copper surface, which in turn reduces the redox potential of copper species from 0.52 V (Cu^+^/Cu vs. SHE) or 0.34 V (Cu^2+^/Cu) to 0.14 V (CuCl/Cu). The most striking difference between the impedance data for the two different corrosive electrolytes is that *R*_ct_ is one to two orders of magnitude higher in sodium chloride than in sulfuric acid for all samples. More importantly, the variation in *R*_ct_ as a function of time was completely different for GPn-Cu compared with B-Cu and P-Cu. B-Cu exhibited an initial steep increase in *R*_ct_, followed by a gradual decrease, with P-Cu exhibiting a similar trend. However, the GPn-Cu *R*_ct_ constantly increased over time. This is a clear indication that the GPn coating slowed the corrosion of copper in the sodium chloride solution. We expect that the GPn acts as both a physical and chemical diffusion barrier, effectively preventing the chloride ions from interacting with the copper surface. The rate of change of *R*_ct_ (d*R*_ct_/d*t*) is plotted as a function of time for each system in Fig. 8. The variation in the GPn-Cu *R*_ct_ was negligible over the entire 50 h in comparison with the control systems.

The impedance data are also presented in Bode plots (Fig. S2). Compared with the Nyquist plots, Bode plots provide a fairly straightforward indication of changes in impedance and, thus, can be used to illustrate the strong potential of the GPn coating as a sensing tool for monitoring the corrosion status of a metal. The Bode plots for GPn-Cu in the two corrosive solutions can be divided into two regions: the high-frequency region of 10^1^ to 10^5^ Hz and the low-frequency region of less than 10^1^ Hz. The high-frequency region appears to be more sensitive to corrosion, with the impedance increasing with exposure time. Calibration curves of log|Z| vs. exposure time at a constant frequency were linear for GPn-Cu, while they were non-linear for P-Cu (Fig. S3). Therefore, the status of the metal in GPn-Cu can be monitored by measuring the time-dependent impedance at a constant frequency.

**Figure 9:**
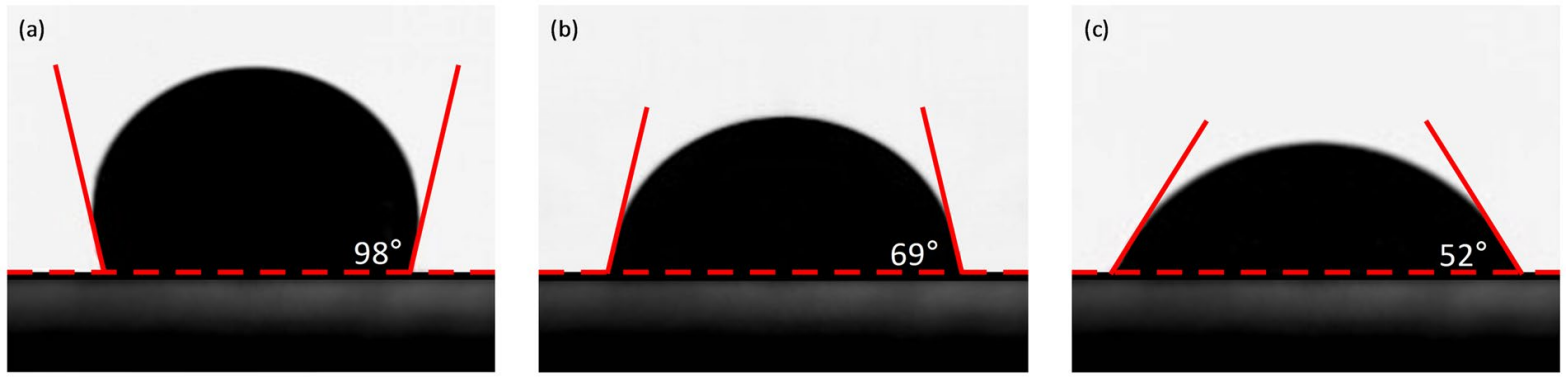
Water-droplet contact-angle test on (**a**) GPn-Cu, (**b**) P-Cu, and (**c**) B-Cu.

The surface nature of the samples was further characterized through contact-angle measurements. The contact angle is a measure of the wettability of the coating layer of the copper. Water contact angles for the different samples are presented in Fig. 9, with GPn-Cu, P-Cu, and B-Cu exhibiting equilibrium contact angles of 98 ± 2°, 69 ± 2°, and 52 ± 3°, respectively, indicating that the GPn coating renders the copper surface hydrophobic, which can be attributed to the microstructural and inherent chemical properties of GPn. All corrosion-related ionic and molecular species are soluble in water and the hydrophobic nature of the GPn layer will contribute to protecting the copper metal in aqueous phases^32^.

**Figure 10:**
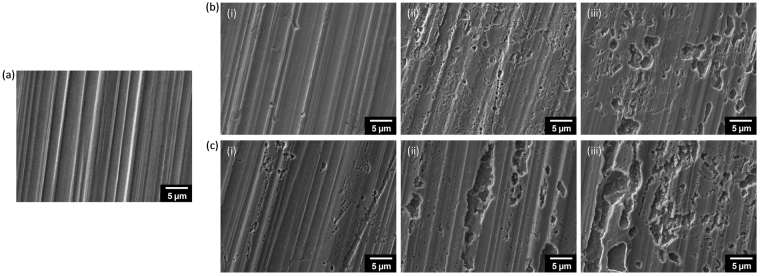
SEM images of the surfaces of (**a**) freshly polished bare copper and (**b**,**c**) (**i**) GPn-Cu, (**ii**) P-Cu, and (iii) B-Cu after immersing in (**b**) 1 M H_2_SO_4_ solution (**c**) and 3.5 wt% NaCl solution (**c**) for 72 h at 25 °C.

Scanning electron microscopy (SEM) was used to directly assess the effect of the nanostructured GPn coating on copper corrosion. The three samples (GPn-Cu, P-Cu, and B-Cu) were exposed to the same two corrosive solutions of sulfuric acid and sodium chloride for 72 h and the coating layer was then peeled off the copper surface prior to SEM observation (Fig. 10). After immersion in the corrosive solutions, the P-Cu and B-Cu copper surfaces possessed many irregular-shaped pits, indicating that the copper was severely damaged by corrosion. It should be noted that the extent of pitting was consistently greatest on the samples immersed in the 3.5 wt% NaCl solution, compared to the 1 M H_2_SO_4_ solution, which would be mainly due to the presence of chloride ions. In contrast to the P-Cu and B-Cu samples, the GPn-Cu surface contained relatively few pits, confirming that the GPn coating provides almost-perfect physical and chemical protection of the copper surface. Similarly, the images of the copper surface indicated that the surface shine of B-Cu disappeared after immersion in the corrosive electrolytes (Fig. S4).

## Conclusions

A GPn was successfully prepared and used to protect copper metal against corrosion in sulfuric acid and sodium chloride solutions. Owing to its conductivity, potentiodynamic polarization and impedance measurements were used to monitor the behavior of the GPn layer as a corrosion barrier. Contact angle and SEM measurements were used to characterize the hydrophobicity of the GPn layer and assess the extent of copper corrosion, respectively. The copper corrosion mechanism was different in the two corrosive solutions. Nevertheless, the GPn coating provided superior corrosion protection, compared with a pure PANI coating, in both solutions. Under induced electrochemical polarization, the protection efficiency of the GPn coating was 17.0% and 20.9% larger than that of pure PANI in 1 M H_2_SO_4_ and 3.5 wt% NaCl solutions, respectively. The GPn can be easily produced using an efficient top-down approach and is adaptable to solution processing^33,34^. Therefore, we believe that commercialization of our material will be possible in the near future. Furthermore, the conductivity of our material renders it suitable for use in developing smart protective layers capable of monitoring the corrosion status of metals.

## Methods

### Materials

Graphite flakes, aniline (≥99.5%), ammonium persulfate (APS) (≥98.0%), NMP (99.5%), (1S)-(+)-10-CSA, 4-DBSA, and D-sorbitol (≥98%) were purchased from Sigma-Aldrich. Sulfuric acid (95.0%), hydrochloric acid (HCl, 35%), ammonium hydroxide solution (NH_4_OH, 25%), and sodium chloride (99.5%) were obtained from OCI Co. Ltd. Copper foil (polished, 30 μm thickness) was purchased from Alfa Aesar.

### Preparation of GPn

First, PANI was synthesized as follows^29–31^: APS (0.10 mol) was dissolved in 1 L of 1 M HCl solution, and aniline (0.44 mol) was dissolved in 1.5 L of 1 M HCl. The two solutions were then mixed and stirred for 90 min at 1.5 °C. The resulting solution was filtered and then washed with excess distilled water and ethanol, which yielded a dark-green filter cake. The dark-green powder was washed several times with 2.5 L of 0.1 M NH_4_OH solution and the resulting product was dried under vacuum at 25 °C. Next, 1 g of the obtained PANI powder was mixed with CSA (3.1 wt%) and DBSA (1.5 wt%) in NMP (92.5 wt%), and undesirable residues or precipitates were removed from the PANI/CSA/DBSA solution. D-sorbitol was dissolved in NMP in a 1:1 weight ratio (PANI/CSA/DBSA-to-sorbitol) at 90 °C, and this solution was mixed with the PANI/CSA/DBSA solution at room temperature.

GPn was then fabricated by exfoliating graphene using the synthesized PANI solution^29,30^. First, 0.5 g of graphite flakes were added to 0.5 L NMP and sonicated for 30 min. The resulting graphene dispersion was added to the PANI solution in a 1:60 graphite:PANI-solution weight ratio, which is the optimized graphene-to-PANI amount for achieving balanced electrical and adhesive properties. The mixed solution was sonicated for 30 min to produce a homogeneous GPn solution. The GPn solution was transferred onto the copper substrate (0.5 mL cm^−2^), which was dried for 5 min under a loading of 4 metric tons at 25 °C. The average coating layer thickness was 15 μm.

### Electrochemical measurements

EIS was performed using a three-electrode cell containing a 1 M H_2_SO_4_ or 3.5 wt% NaCl solution as the electrolyte, with a Pt auxiliary electrode and a Ag/AgCl reference electrode. All electrochemical measurements were performed using a Metrohm Autolab B.V. PGSTAT101 potentiostat/galvanostat. The EIS equivalent circuits and component values were calculated using NOVA 2.1.2 software.

### Characterization

SEM was performed using a JEOL JSM-7500F microscope. Contact angle measurements were conducted using a SurfaceTech GSA-X goniometer.

## Electronic supplementary material

Supplementray Information
