# Supplementary material for: A Solution-Processable, Nanostructured, and Conductive Graphene/Polyaniline Hybrid Coating for Metal-Corrosion Protection and Monitoring
Source: Sci Rep. 2017 Nov 9;7:15184. doi: 10.1038/s41598-017-15552-w (PMC5680262; doi:10.1038/s41598-017-15552-w)
Supplement: Supplementary file 1 — Supplementray Information [file 41598_2017_15552_MOESM1_ESM.pdf]

Supporting Information

A Solution-Processable, Nanostructured, and Conductive Graphene/Polyaniline Hybrid Coating for Metal-Corrosion Protection and Monitoring

Saerona Kim,[2] Thanh-Hai Le,[2] Chul Soon Park,[2] Geunsu Park,[2] Kyung Ho Kim,[2] Semin Kim,[2] Oh Seok Kwon,[3] Gyun Taek Lim,[1,2,*] and Hyeonseok Yoon[1,2,*]

[1]School of Polymer Science and Engineering, Chonnam National University, 77 Yongbong-ro, Buk-gu, Gwangju 61186, South Korea.

[2]Department of Polymer Engineering, Graduate School, Chonnam National University, 77 Yongbong-ro, Buk-gu, Gwangju 61186, South Korea.

[3]BioNanotechnology Research Center, Korea Research Institute of Bioscience and Biotechnology (KRIBB), 125 Gwahak-ro, Yuseong-gu, Daejeon 34141, South Korea

Corresponding Authors:

*E-mail: gtlim@chonnam.ac.kr, hyoon@chonnam.ac.kr

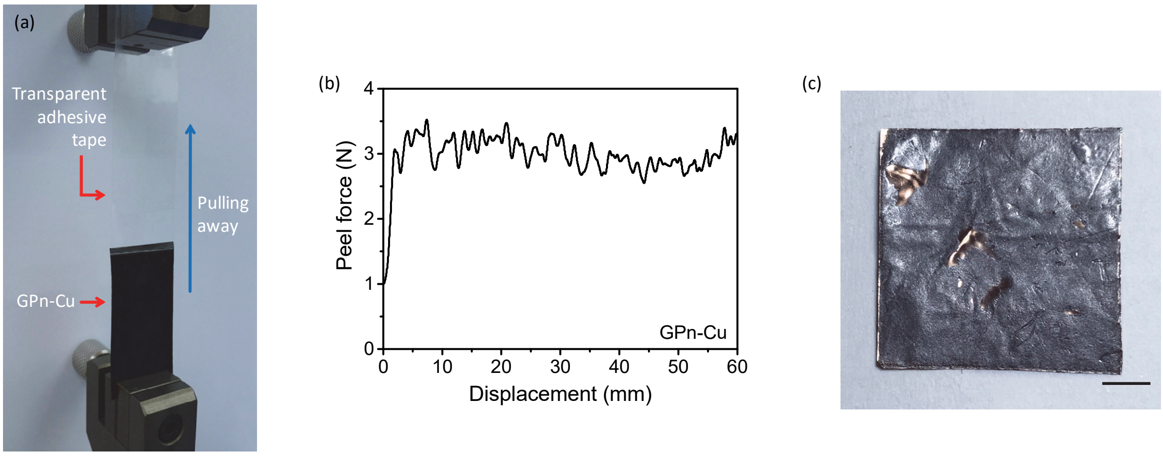

Figure S1. Mechanical adhesion testing of GPn coating: (a) Photograph illustrating a peel test, and (b) force-distance curve for GPn-Cu with the adhesive tape. (c) Photo of graphene_only coated copper as a control (the scale bar is 3 mm).

The adhesion test was performed with a 180° peeling geometry at 1 mm s^{-1} using a Universal Testing Machine STM-5, in which transparent 3M Scotch 600 adhesive peeling tape was used, and the specimen size was 25 mm × 60 mm. The specimen was composed of two parts: a plate (copper foil) and a coating (GPn). The area of both the copper foil and GPn coating layer was 25 mm × 60 mm.

As seen in Figure S1a, the test was performed by peeling back the tape at an angle of 180° from the GPn-Cu specimen. No visible GPn debris was observed on the detached tape, indicating that the GPn stuck well to the copper and did not peel. The average calculated peel force was as high as ~3 N during testing (Figure S1b). This strong adhesive strength is essential for practical coating applications. As seen in Figure S1c, the adhesion of graphene_only coating on copper was poor.

Table S1. Equivalent-circuit component values for GPn-Cu calculated through Figure 4a impedance-plot fitting.

Time (h)	R_s (Ω)	R_{ct} (Ω)	C_{dl} (F)	C_{cl} (F)
0	1.62	0.13	3.67×10^{-3}	6.88×10^{-3}
2	1.61	0.15	2.96×10^{-3}	7.52×10^{-3}
4	1.66	0.16	2.24×10^{-3}	7.59×10^{-3}
6	1.67	0.18	1.73×10^{-3}	7.51×10^{-3}
8	1.68	0.19	1.29×10^{-3}	7.48×10^{-3}
24	1.72	0.47	1.19×10^{-4}	6.07×10^{-3}
48	1.75	0.95	4.04×10^{-5}	5.04×10^{-3}

Table S2. Equivalent-circuit component values for P-Cu calculated through Figure 4b impedance-plot fitting.

Time (h)	R_s (Ω)	R_{ct} (Ω)	C_{dl} (F)	CPE (F)
0	1.41	9.64	1.54×10^{-5}	4.13×10^{-2}
2	1.24	24.45	1.09×10^{-5}	1.58×10^{-2}
4	1.18	30.19	1.02×10^{-5}	1.34×10^{-2}
6	1.17	34.12	9.62×10^{-6}	1.12×10^{-2}
8	1.21	36.33	9.44×10^{-6}	1.02×10^{-2}
24	1.29	52.07	8.32×10^{-6}	7.35×10^{-3}
48	1.23	67.42	7.79×10^{-6}	6.55×10^{-3}

Table S3. Equivalent-circuit component values for B-Cu calculated through Figure 4c impedance-plot fitting.

Time (h)	R_s (Ω)	R_{ct} (Ω)	CPE (F)	W (mMho $s^{1/2}$)
0	1.24	538.27	4.59×10^{-5}	3.74
2	1.22	262.25	5.15×10^{-5}	10.50
4	1.21	188.29	4.58×10^{-5}	11.30
6	1.21	141.90	4.98×10^{-5}	11.73
8	1.24	140.72	5.21×10^{-5}	16.73
24	1.27	140.27	4.97×10^{-5}	16.90
48	1.37	108.97	6.12×10^{-5}	23.55

Table S4. Equivalent-circuit component values for GPn-Cu calculated through Figure 4d impedance-plot fitting.

Time (h)	R_s (Ω)	R_{ct} (Ω)	CPE (F)	C_{cl} (F)
0	6.12	24.96	1.79×10^{-2}	3.39×10^{-3}
2	5.72	25.89	1.53×10^{-2}	3.15×10^{-3}
4	5.93	33.36	1.39×10^{-2}	3.11×10^{-3}
6	6.20	40.66	1.21×10^{-2}	2.72×10^{-3}
8	6.53	44.67	1.11×10^{-2}	2.21×10^{-3}
24	8.23	56.25	6.97×10^{-3}	1.82×10^{-3}
48	9.81	73.07	5.80×10^{-3}	0.42×10^{-3}

Table S5. Equivalent-circuit component values for P-Cu calculated through Figure 4e impedance-plot fitting.

Time (h)	R_s (Ω)	R_{ct} (Ω)	CPE (F)	C_{cl} (F)	W (mMho $s^{1/2}$)
0	7.43	68.55	2.98×10^{-4}	1.46×10^{-4}	-
2	7.63	125.80	4.90×10^{-4}	1.19×10^{-4}	-
4	7.74	145.81	4.85×10^{-3}	1.18×10^{-4}	1.18
6	7.99	159.60	3.78×10^{-3}	1.34×10^{-4}	1.13
8	8.07	161.76	3.43×10^{-3}	1.27×10^{-4}	1.15
24	11.75	30.93	7.61×10^{-3}	1.05×10^{-3}	3.64
48	15.52	27.57	7.98×10^{-3}	2.12×10^{-3}	3.85

Table S6. Equivalent-circuit component values for B-Cu calculated through Figure 4f impedance-plot fitting.

Time (h)	R_s (Ω)	R_{ct} (Ω)	CPE (F)	W (mMho $s^{1/2}$)
0	6.02	1428.38	6.51×10^{-4}	0.64
2	6.02	3813.80	2.45×10^{-4}	0.24
4	6.02	3721.61	0.57×10^{-3}	0.22
6	5.87	3655.26	0.61×10^{-3}	0.21
8	5.72	3624.90	0.63×10^{-3}	0.22
24	6.12	3416.57	1.13×10^{-3}	0.23
48	6.06	2564.32	3.72×10^{-3}	0.31

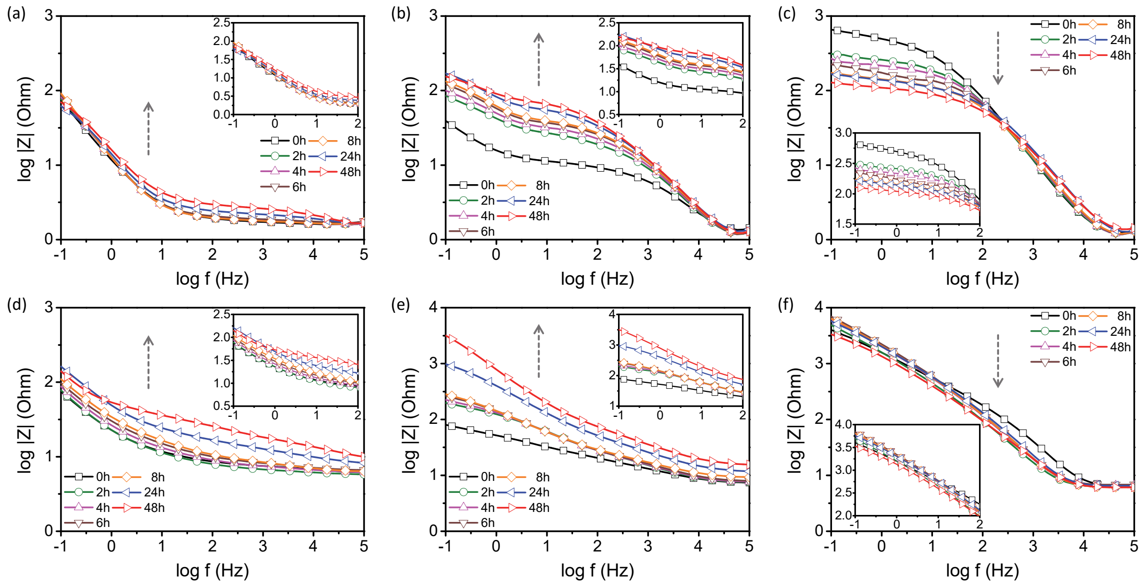

Figure S2. Bode plots for (a, d) GPn-Cu, (b, e) P-Cu, and (c, f) B-Cu measured in (a-c) 1 M H_2SO_4 solution and (d-f) 3.5 wt% NaCl solution. The insets show magnified plots of the high-frequency regions.

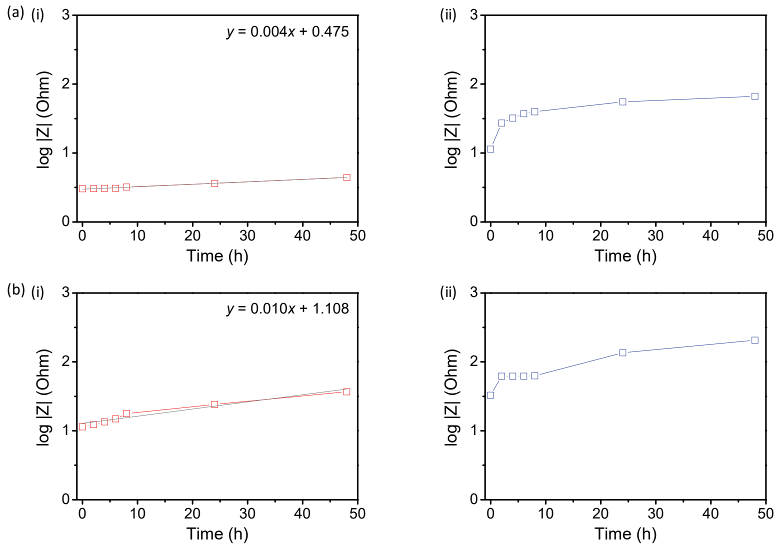

Figure S3. Plots of log |Z| vs. exposure time at a constant frequency of log f = 1 for (i) GPn-Cu and (ii) P-Cu. The log |Z| values were calculated from Figure S2. (a) 1 M H_2SO_4 solution and (b) 3.5 wt% NaCl solution.

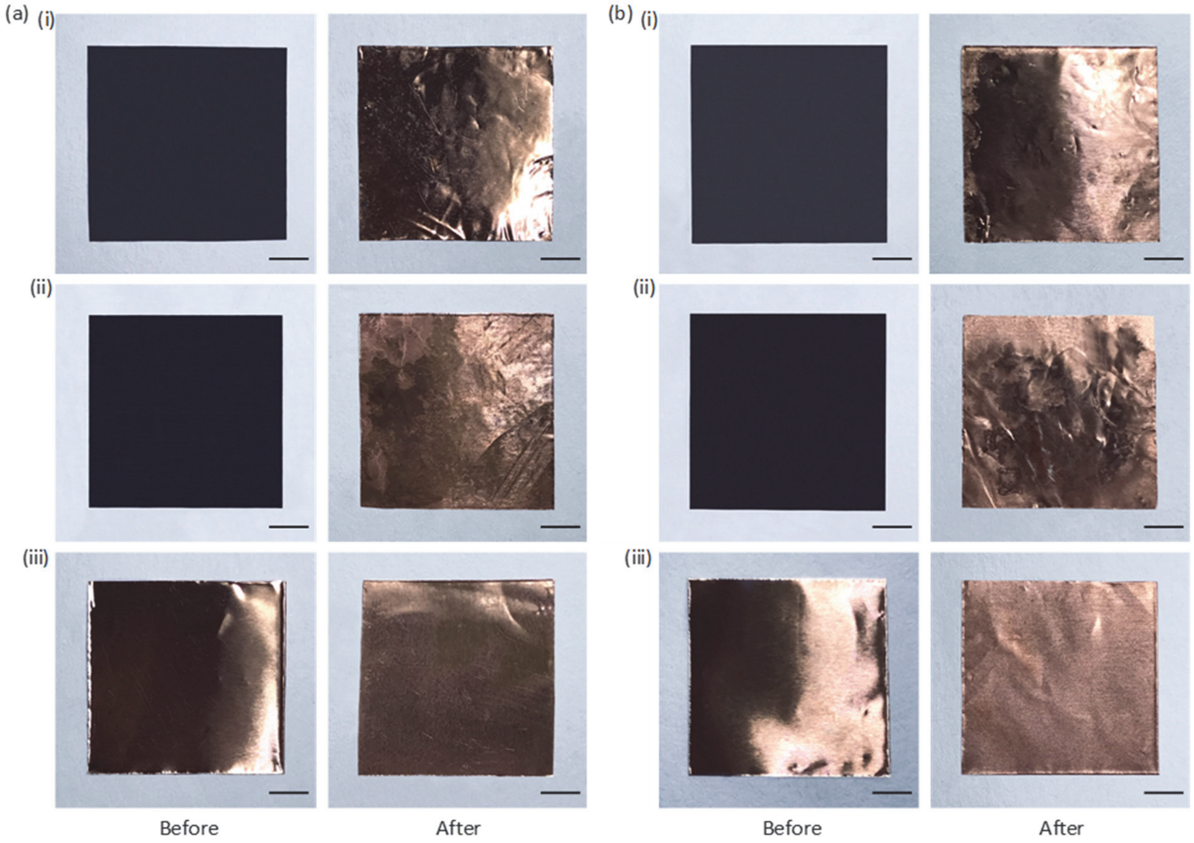

Figure S4. Photos of (i) GPn-Cu, (ii) P-Cu, and (iii) B-Cu before and after immersion in (a) 1 M H_2SO_4 solution and (b) 3.5 wt% NaCl solution for 72 h at 25 °C (the scale bar is 3 mm). The coating layer was peeled off from the copper surface after the immersion for observation.
